# A 3,000-year-old, basal *S*. *enterica* lineage from Bronze Age Xinjiang suggests spread along the Proto-Silk Road

**DOI:** 10.1371/journal.ppat.1009886

**Published:** 2021-09-21

**Authors:** Xiyan Wu, Chao Ning, Felix M. Key, Aida Andrades Valtueña, Aditya Kumar Lankapalli, Shizhu Gao, Xuan Yang, Fan Zhang, Linlin Liu, Zhongzhi Nie, Jian Ma, Johannes Krause, Alexander Herbig, Yinqiu Cui

**Affiliations:** 1 School of Life Sciences, Jilin University, Changchun, China; 2 School of History and Culture, Henan University, Kaifeng, China; 3 Max Planck Institute for the Science of Human History, Jena, Germany; 4 Max Planck Institute for Infection Biology, Berlin, Germany; 5 Max Planck Institute for Evolutionary Anthropology, Leipzig, Germany; 6 College of Pharmacia Sciences, Jilin University, Changchun, China; 7 Department of Radiation Oncology, The Second Hospital of Jilin University, Changchun, China; 8 Research Center for Chinese Frontier Archaeology, Jilin University, Changchun, China; 9 School of Cultural Heritage, Northwest University, Xi’an, China; 10 Key Laboratory for Evolution of Past Life and Environment in Northeast Asia (Jilin University), Ministry of Education, Changchun, China; University of California, Davis, UNITED STATES

## Abstract

*Salmonella enterica* (*S*. *enterica*) has infected humans for a long time, but its evolutionary history and geographic spread across Eurasia is still poorly understood. Here, we screened for pathogen DNA in 14 ancient individuals from the Bronze Age Quanergou cemetery (XBQ), Xinjiang, China. In 6 individuals we detected *S*. *enterica*. We reconstructed *S*. *enterica* genomes from those individuals, which form a previously undetected phylogenetic branch basal to Paratyphi C, Typhisuis and Choleraesuis–the so-called Para C lineage. Based on pseudogene frequency, our analysis suggests that the ancient *S*. *enterica* strains were not host adapted. One genome, however, harbors the *Salmonella* pathogenicity island 7 (SPI-7), which is thought to be involved in (para)typhoid disease in humans. This offers first evidence that SPI-7 was acquired prior to the emergence of human-adapted Paratyphi C around 1,000 years ago. Altogether, our results show that *Salmonella enterica* infected humans in Eastern Eurasia at least 3,000 years ago, and provide the first ancient DNA evidence for the spread of a pathogen along the Proto-Silk Road.

## Author summary

Recent studies that use DNA extracted from ancient human remains have shown that the bacterial pathogen *Salmonella enterica* has infected humans since prehistoric times. Expanding the knowledge about its geographic spread and genetic diversity we reconstructed six ancient *Salmonella* genomes from individuals excavated at the Bronze Age Quanergou cemetery in Xinjiang, China. Our analysis shows that this form of *Salmonella* was closely related to a type that today specifically infects humans and/or pigs. However, based on our genetic assessment we determined that our ancient strains were not host adapted yet. Furthermore, we could show that in the course of its evolution the bacterium acquired some important virulence factors earlier than previously thought. Interpreting our results within an archaeological context we suggest that in Bronze Age Eastern Eurasia the spread of this pathogen was likely promoted through trade networks referred to as the Proto-Silk Road.

## Introduction

*Salmonella enterica* (*S*. *enterica*) is a Gram-negative pathogenic bacterium that can infect a wide variety of warm-blooded hosts and causes enteric fever and gastroenteritis [1]. On a global scale, at least 170 million cases of salmonellosis occur each year, resulting in up to 1 million deaths [2,3]. Traditionally, its diversity has been described based on serological differences, and approximately 2,600 *Salmonella* serovars have been identified so far [4]. Despite its worldwide distribution today, little is known about its spread and contribution to prehistoric epidemic events.

Ancient pathogen genomes are crucial for studying pathogen evolution, calibrating the molecular clock and providing time transect data to illustrate phylogenetic diversity [5]. With the development of ancient DNA technologies, pathogen genomes can be retrieved from archaeological remains, which enables us to reconstruct the phylogenetic history of pathogens and identify the causative agents of past epidemic events. Human-adapted Paratyphi C has been reconstructed from a 13th-century skeleton from Norway, providing direct evidence for the presence of this lineage in Europe about 800 years ago [6]. Vågene et al. recovered Paratyphi C genomes from a 16th-century colonial era Mexican site suggesting that this pathogen may have contributed to the collapse of native populations after its introduction to the New World [7]. Key et al. recovered eight diverse ancient *S*. *enterica* genomes across Western Eurasia, suggesting that the emergence of human-adapted *S*. *enterica* Paratyophi C was linked to the Neolithization process [8]. However, whether and when *Salmonella* had appeared in Eastern Eurasia or where and how the human-adapted Paratyphi C evolved are still largely unknown.

Geographically located in northwestern China and part of the “Silk Road”, Xinjiang has been a hub connecting eastern and western Eurasia since the Bronze Age [9]. Starting from the early Bronze Age, the Eurasian Steppe (ES) had witnessed several major cultural changes and large-scale population movements [10–13]. Millet of East Asian origin spread westward into Europe, and conversely, wheat and barley of the Near East origin spread eastward into East Asia, perhaps via what was known as the Inner Asian Mountain Corridor along the Tianshan Mountains in Xinjiang [14]. The ES pastoralists may have served as an important agent for such cereal globalization [15–17]. Furthermore, DNA studies of ancient human remains from Xinjiang suggested that populations in this region were already admixed between eastern and western Eurasians since the Bronze Age [18–20], pointing out that the Xinjiang region had served as a main corridor for trans-Eurasian contacts and likewise the transmission of certain pathogens [21,22].

**Fig 1:**
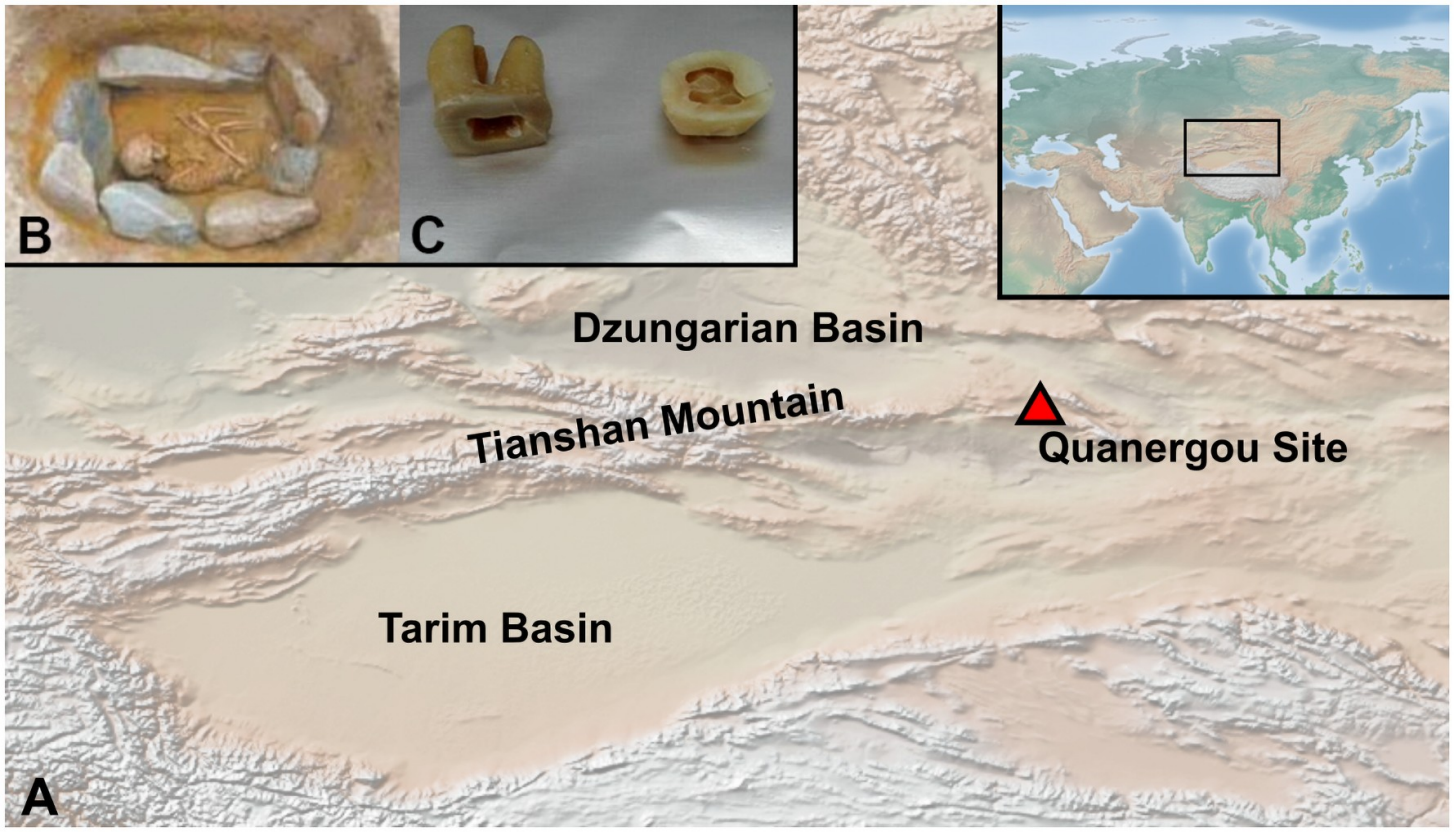
Geographical origin and depiction of archaeological samples. **A.** Geographical location of the XBQ site in this study. Map made with Natural Earth (https://www.naturalearthdata.com/downloads/50m-raster-data/50m-cross-blend-hypso/). **B.** A child buried in a stone coffin. **C.** The tooth was sectioned at the cementoenamel junction and a sample was drilled from the crown pulp chamber.

The XBQ site is located in Xinjiang, to the eastern part of the Tianshan Mountains (Fig 1). The cemetery was dated to the Bronze Age and likely used by mobile pastoralists [23]. Multiple simultaneous burials were excavated from the cemetery, showing a high mortality rate for young adults and in particular children and infants without obvious skeletal trauma [23,24], which has not been observed elsewhere in the region [25]. Until now, no consensus has been reached among archaeologists on how to explain this unusual phenomenon. Here we present ancient DNA evidence for *S*. *enterica* infection in multiple individuals from XBQ suggesting systemic infectious disease that might have traveled along the Proto-Silk Road (the connection routes between the western and eastern Eurasia that preceded the Silk Road) and contributed to the suddenly high mortality.

## Results

### Identification of *S*. *enterica* in the Bronze Age XBQ individuals

We screened 0.4–25 million raw shotgun sequencing reads generated from dental pulp of 14 individuals recovered from the XBQ site in Xinjiang, China (see Materials and methods) to assess the possible presence of pathogen DNA. The results were evaluated by the edit distance distribution, which represented the number of nucleotide positions in a mapped DNA sequence that differ from the reference that it aligns to. [26,27]. As a result, 6 XBQ individuals showed low edit distance of the aligned reads indicating high sequence similarity with *S*. *enterica* (S1 Fig). Between 18 to 2,379 sequencing reads from these individuals were assigned to *S*. *enterica* with the two related serovars Paratyphi C and Choleraesuis as the best match. Besides, we assessed DNA deamination patterns for assigned reads. C-to-T substitutions from the 5’ end and G-to-A substitutions from the 3’ end were present in 6 positive samples (S2 Fig), suggesting the authenticity of our results.

### Reconstruction of the ancient *S*. *enterica* genomes

In order to retrieve full *S*. *enterica* genomes from the XBQ individuals, we performed in-solution hybridization capture to enrich the sequencing libraries for *S*. *enterica* DNA fragments. We generated between 13.4 to 28.37 million raw reads for the 6 positive samples (XBQM20, XBQM90, XBQM11, XBQM7, XBQM83 and XBQM64). After mapping to the Paratyphi C RKS4594 genome (NC_012125.1), the data showed a clear DNA deamination pattern consistent with an ancient origin [28] (S3 Fig).

**Fig 2:**
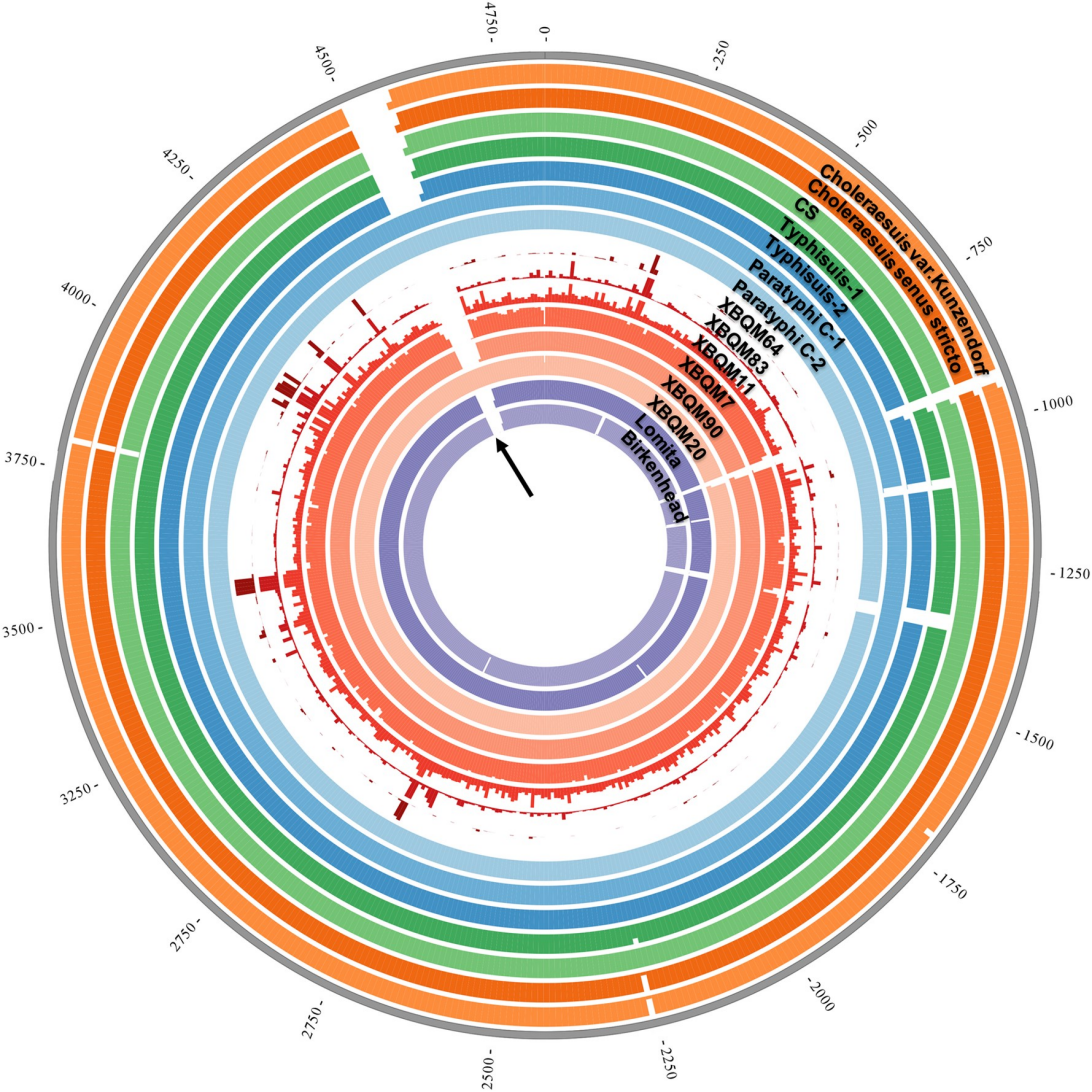
Coverage overview of the modern Para C and XBQ genomes. The average coverage was calculated for 10kb regions for the chromosome, each ring represents a maximum of 5X coverage. The figure was generated with Circos [80]. The black arrow represents the position of SPI-7.

To generate high quality *S*. *enterica* genomes and remove deaminated cytosines owing to ancient DNA damages, we built additional libraries with uracil DNA glycosylase (UDG) treatment (see Materials and methods), which were also subjected to capture and a total of 8.83–96.36 million raw reads were generated (Table 1). Furthermore, we evaluated the in-solution capture efficiency based on the percentage of reads on target among all sequenced reads (endogenous DNA), the proportion of genome covered and the significant differences in the mean coverage (S1 Table). The percentage of endogenous DNA of XBQM20, XBQM90, XBQM11, XBQM7, XBQM83 and XBQM64 were estimated to be 228-, 104-, 80-, 118-, 41- and 73-fold higher after the in-solution capture, respectively, resulting in an average genome-wide coverage of 0.09X to 4.2X and a proportion of the genome covered of 3.69% to 86.83% (Fig 2 and S1 Table).

**Table 1:** Statistics of the *Salmonella enterica* Genome Reconstruction (including UDG library and Non-UDG library) of the XBQ individuals.

Sample Name	Tissue Sampled	C14 dating (BP)	Library treatment	Merged reads	Mapped reads	Endogenous DNA (%)	Mean coverage	Average fragment length
XBQM20	Dental pulp	3050	Non-UDG	28372090	549836	1.938	0.6437	81.89
XBQM20	Dental pulp	3050	UDG	52625782	3875319	7.364	3.2664	67.12
XBQM90	Dental pulp	3070 to 2920	Non-UDG	23353937	485815	2.08	0.3467	106.34
XBQM90	Dental pulp	3070 to 2920	UDG	58928968	2330756	3.955	3.8519	54.26
XBQM7	Dental pulp	3210 to 3000	Non-UDG	13403597	164778	1.299	0.1979	64.75
XBQM7	Dental pulp	3210 to 3000	UDG	96358257	1195832	1.157	0.9265	54.1
XBQM83	Dental pulp	NA	Non-UDG	13827321	146472	1.059	0.0732	60.47
XBQM83	Dental pulp	NA	UDG	59022296	364829	0.618	0.2639	51.61
XBQM11	Dental pulp	3160 to 2960	Non-UDG	16362465	276052	1.687	0.2349	63.43
XBQM11	Dental pulp	3160 to 2960	UDG	8825015	106783	1.21	0.4798	52.22
XBQM64	Dental pulp	NA	Non-UDG	14806257	86420	0.584	0.0299	86.23
XBQM64	Dental pulp	NA	UDG	16991502	76625	0.451	0.0651	58.57

Notes: BP, before present; Samples without appropriate radiocarbon dating are marked with NA

### Ancient *S*. *enterica* genomes form a previously uncharacterized branch and reveal early diversification under the Para C lineage

**Fig 3:**
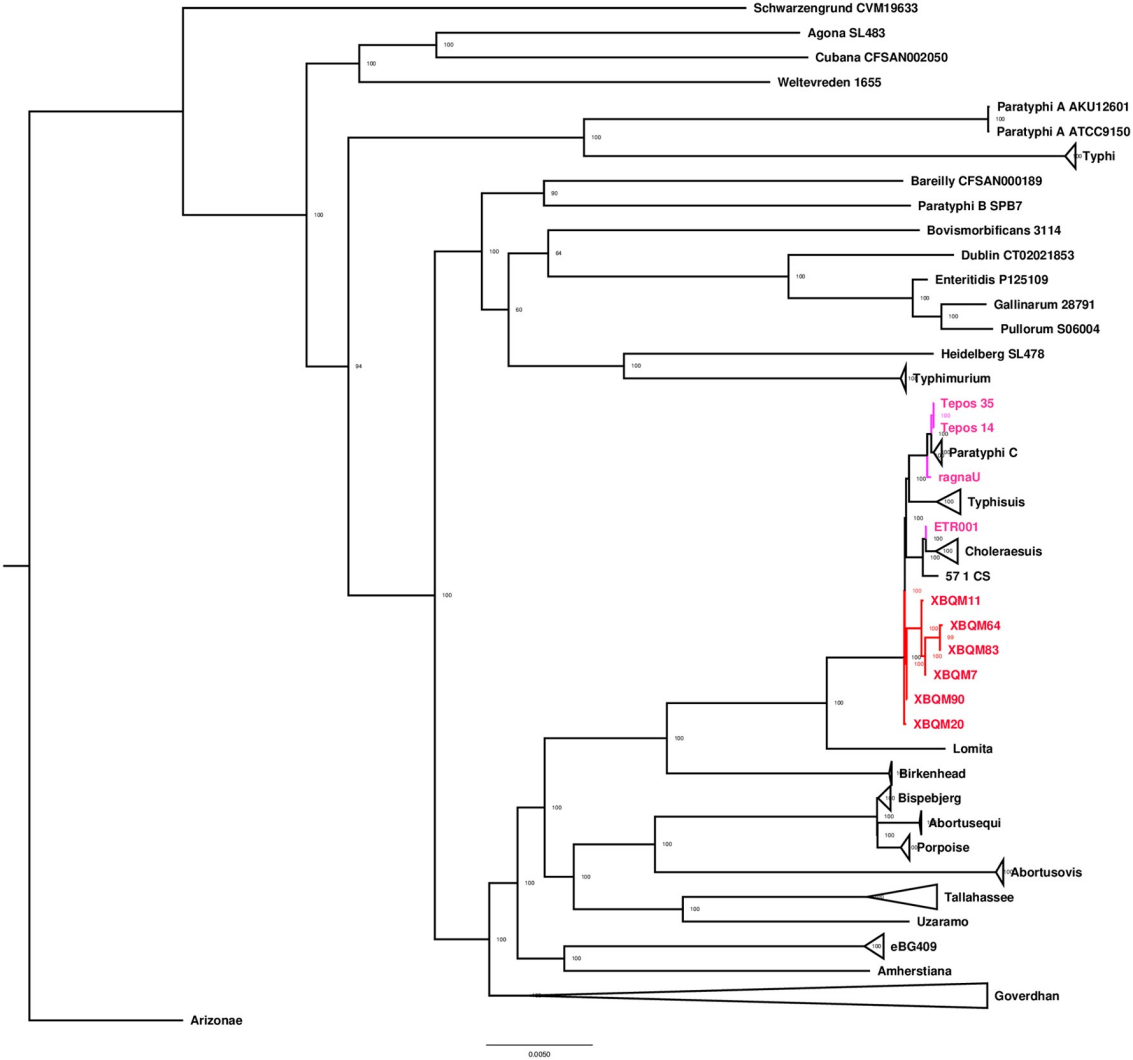
Phylogenetic relationships of the ancient and modern *Salmonella enterica*. A maximum likelihood tree was generated with 1000 replicates. Part of modern genomes are collapsed based on their predicted serovar in order to better representation of the relevant information, Arizonae strain is used as the outgroup. Ancient genomes reported in this study are shown in red, and previously reported ancient genomes (Tepos35, Tepos14, ragnaU and ETR001) in pink. Most of ancient XBQ genomes form a unique branch basal to the Para C lineage.

To determine the phylogeny of our ancient *S*. *enterica* strains, we mapped them together with previously published ancient and modern *S*. *enterica* strains to the Paratyphi C RKS4594 reference genome using strict filtering parameters (see Materials and methods). A total of 485 genomes were included to build the phylogenetic tree (including four previously published ancient genomes: Tepos14, Tepos35 [7], ragnaU [6] and ETR001 [8], 475 modern genomes as well as the six samples from this study). Given that our genomes are of relatively low genomic coverage, we evaluated SNP calls by applying the tool SNPEvaluation to prevent false positive SNP (see Materials and methods) [29]. 241,039 SNPs that passed our evaluation criteria were used to build a Maximum Likelihood (ML) tree implemented in IQ-Tree 1.6.12 with 1000 bootstrap replicates [30]. Most of XBQ genomes form a single clade and fall very close to the node that is basal to the serovars Paratyphi C, Typhisuis and Choleraesuis, all of which belong to the Para C lineage [6] (Fig 3).

**Fig 4:**
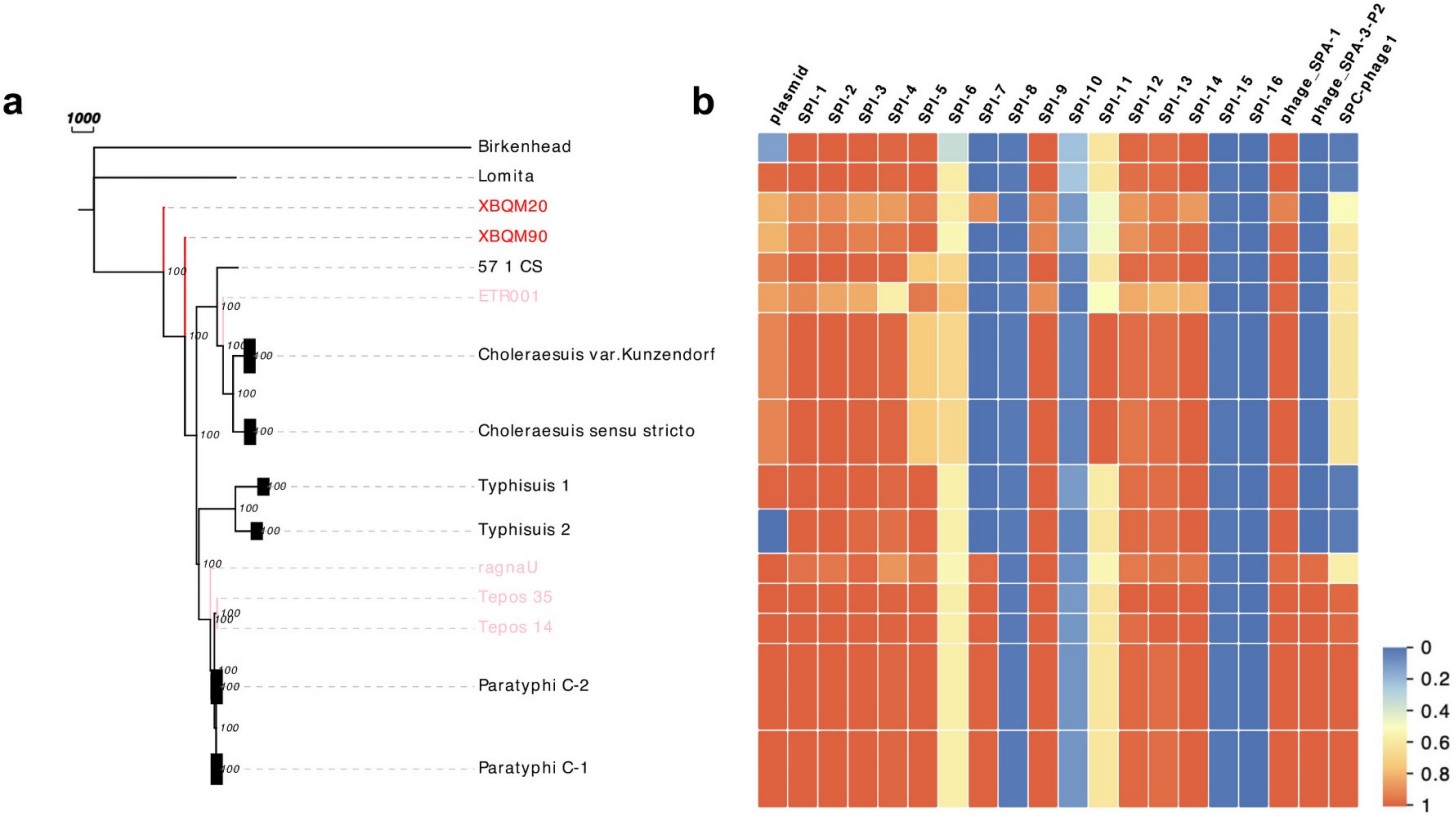
Phylogenetic tree of the Para C lineage and the gain and loss of virulence factors. **A.** Maximum parsimony tree of the Para C lineage. Paratyphi C, Typhisuis and Choleraesuis strains are collapsed based on their predicted serovar in order to better represent the relevant information and the Birkenhead serovar was used as the outgroup. **B.** Covered percentage of *Salmonella* virulence factors including the pSPCV plasmid, *Salmonella* pathogenicity islands (SPI1-SPI16) and prophages. For grouped strains, such as Paratyphi, Typhisuis and Choleraesuis, we showed the average percentage of covered region.

In order to construct a more detailed phylogeny of the Para C lineage, we rebuilt the phylogenetic tree restricting our analysis to genomes under the Para C lineage. The final dataset includes 219 modern Para C lineage strains, 4 previously published ancient genomes [6–8] as well as two genomes (XBQM20 and XBQM90) from this study with an average coverage of at least 3X. In comparison to other genomes, XBQM20 and XBQM90 form a unique branch with XBQM20 falling basal to XBQM90. The relatively short branch lengths of XBQM20 and XBQM90 indicate a high genetic similarity to the direct progenitor of Paratyphi C, Typhisuis and Choleraesuis (Fig 4A). Given that the conventional phylogenetic analysis on low coverage data could produce biased results, we also adopted an alternative approach of phylogenetic placement (MGplacer) [31], whereby our ancient genomes were placed on a fixed Maximum Likelihood tree constructed by modern Para C lineage strains. As a result, the branch node of our ancient genomes was placed basal to the serovars Paratyphi C, Typhisuis and Choleraesuis, which is consistent with the phylogeny results by IQtree (S4 Fig).

### Major pathogenicity island SPI-7 is present in XBQM20

It has been proposed that the *Salmonella* pathogenicity islands (SPI), the virulence plasmid and prophage are associated with differences in clinical manifestations and virulence of *S*. *enterica* infection [32]. We analyzed the presence/absence patterns for genes in these genomic regions restricting our analyses to XBQM20 and XBQM90. Our results are in line with previous reports [6,8], which the ancient and modern strains largely show genomic stability, however, with a notable exception of SPI-7 (Fig 4B). SPI-7 encodes a capsular polysaccharide that is used to shield against the host immune system and is associated with typhoid fever [33]. Previous studies showed that SPI-7 was present in modern and ancient Paratyphi C strains but absent elsewhere in the Para C Lineage [6,7]. Interestingly, we find SPI-7 is present in XBQM20 (S5 Fig), while absent in XBQM90 (Fig 2), which shows the presence of SPI-7 within a non-Paratyphi C serovar part of the Para C Lineage that likely affected its pathogenicity (Fig 4).

### Estimation of divergence time

The newly reported ancient genomes here allow us to refine previously reported molecular dates for the diversification of Paratyphi C, Typhisuis and Choleraesuis. We assessed the temporal signal using TempEst [34] and the calculated correlation coefficient R^2^ value was 0.2368, which showed a sufficient temporal signal and permitted us to proceed with molecular dating analysis. Plots of the root-to-tip regression of genetic distances and sample ages are shown in S6 Fig. To estimate the time to the Most Recent Common Ancestor (tMRCA) of all Para C lineage strains, we employed the coalescent Bayesian skyline models implemented in BEAST v1.10.1 [35], with a relaxed lognormal molecular clock and the General Time Reversible model (GTR) with six gamma categories (see Materials and methods). Here, it produced a coalescent date of 3,185 years ago (95% HPD: 3,088–3,219) for the split of our ancient samples and Paratyphi C, Typhisuis plus Choleraesuis (S7 Fig), while a previous estimated tMRCA for Paratyphi C, Typhisuis and Choleraesuis was 3,428 years ago (95% Cl,1,707–6,142BP) [6]. Additional posterior estimates for the main internal node dates were summarized in the supplementary information (S2 Table). Overall, our inferred molecular dates are more confined.

### Inference of host specificity using pseudogene frequency

**Fig 5:**
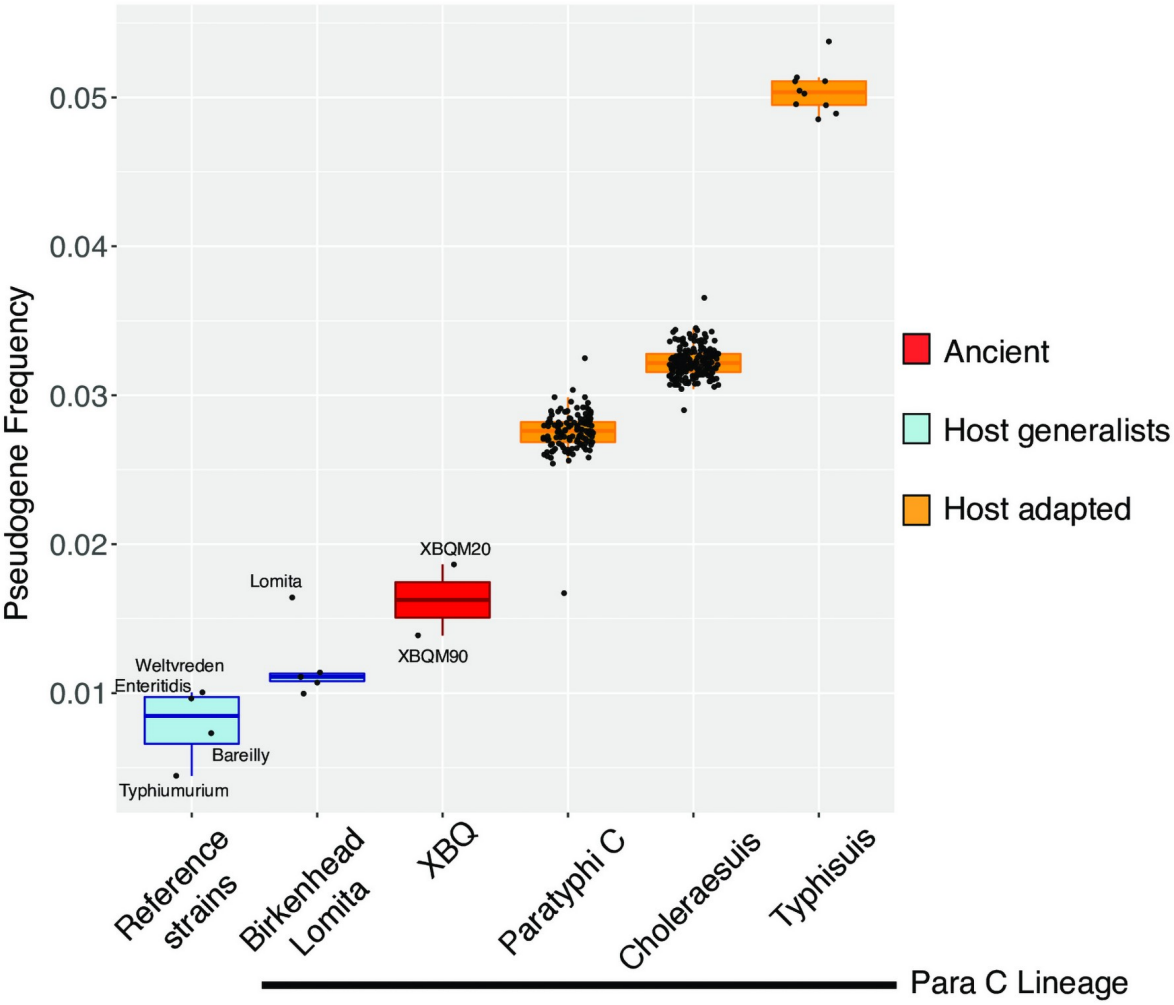
Pseudogene frequency of reference strains, XBQ samples, and strains from the Para C lineage. Relative frequency of pseudogenes for known host generalists (blue), ancient XBQ samples (red), and known host adapted strains part of the Para C Lineage (orange). XBQ genomes with genome-wide coverage above 3X were included. Included reference strains (incl. BioSample identifier) not part of the Para C lineage are: Typhimurium (SAMN03996249), Weltevreden (SAMEA1904377), Bareilly (SAMN01823701), Enteritidis (SAMEA1705941).

It has been recognized that pseudogene accumulation is related to the host specificity in *S*. *enterica* [8,36]. In order to understand the host specificity of our ancient strains, we analyzed the frequency of pseudogenes based on frameshift mutations and premature stop mutations, which we compare to modern as well as previously published ancient genomes [8]. In total, 1,717 genes and 32 pseudogenes were contained in XBQM20 and 2,522 genes and 35 pseudogenes were contained in XBQM90 (S3 Table). Only 8 pseudogenes (~25%) were shared between both ancient genomes, pointing to continuous acquisition of pseudogenes since their most recent common ancestor. Overall, we observe that at least 41–61% of pseudogenes are shared among genomes within each serovar (S8 Fig), confirming that pseudogenization occurred before and after serovar divergence. The pseudogene frequency of our ancient *Salmonella* was in-between the observed frequency in host generalists and host adapted serovars (Fig 5), which is in line with their phylogenetic placement basal to host adapted Paratyphi C (human), Choleraesuis (human/pig), and Typhisuis (pig). Thus, our data suggests that the ancient *S*. *enterica* serovars found in XBQ population were not confined to infect humans.

## Discussion

In the past decades, the study of paleopathology had largely relied on the historical reporting and morphological studies of human remains to characterize ancient epidemics events. With the development of next generation sequencing technologies [37] and the application of DNA enrichment methods [38], DNA sequences can now be retrieved from ancient human remains to verify the causative agent of diseases that cannot be achieved by other means [27,39]. More than 200 simultaneous burials were found in XBQ, as well as disproportional mortality of children. The absence of skeletal trauma suggests that they were likely not killed by violence or wars. Physical anthropological analyses showed a high mortality rate of juveniles and infants in XBQ compared with other contemporaneous cemeteries in same region [25,40]. Many archaeologists suspected this kind of unusual phenomenon could be caused by some kind of infectious diseases, but so far, no concrete conclusion could be made. Here, we used molecular methods to screen for pathogens in ancient human remains from XBQ. Six out of fourteen individuals were identified to carry detectable *S*. *enterica* DNA, providing an explanation for the unusual mortality event. Considering that *Salmonella* is mainly transmitted due to the intake of contaminated food and water, our findings could reflect the poor hygiene in the XBQ region such as consumption of unprocessed contaminated foods (such as meat or milk products) and water, contact with infected feces, as well as infective animals or humans.

Here we present the first evidence that *Salmonella* infected humans in East Asia roughly 3,000 years ago, which predates definite historical recordings of epidemics in China [41,42]. This further underlines that the study of ancient pathogens has great potential to track epidemic events in ancient times. In addition, all ancient genomes in this study, except for XBQM20, show an absence of SPI-7, which encodes the Vi capsular polysaccharide that could promote enteric fever in humans [33]. The presence of SPI-7 region in XBQM20 indicates that this *S*. *enterica* strain could have caused a systemic paratyphoid-like infection, while the XBQM90 strain might only have caused non-typhoidal salmonellosis due to the absence of SPI-7 [43]. Previous studies have suggested that SPI-7 was acquired prior to the diversification of Paratyphi C and is associated with specificity to the human host [6]. However, in our study we found the acquisition of SPI-7 was earlier than previous thought, suggesting that SPI-7 was acquired prior to the divergence of Paratyphi C and Choleraesuis. Its subsequent loss in parts of the Para C diversity could have been related to host adaptation processes.

Despite the large genetic diversity of *S*. *enterica* observed today, all ancient genomes reported so far are confined within the Para C Lineage or a broader group of *S*. *enterica*, the so-called “Ancient Eurasian Super Branch” [8], which further suggests a far-reaching global spread of those *S*. *enterica* serovars during prehistoric times. Although all the ancient strains in this study represent a good proxy of the direct progenitors of today’s Para C diversity, they also revealed genetic heterogeneity among each other. Firstly, XBQM20 forms a distinct branch with respect to the other XBQ samples in the phylogeny analysis (Fig 3). Secondly, we identified the presence of SPI-7 in XBQM20, but not in XBQM90 (Fig 2). Thirdly, we found there were private SNPs in our ancient samples which have passed our filtering criteria (S4 and S5 Tables). These differences indicate a genetic diversity among our ancient genomes, which suggests that multiple distinct strains were causing infections in the human population at the same time. The genomic heterogeneity of *Salmonella* observed in XBQ indicate possible recurrent epidemic events, which could imply multiple introductions of this pathogen into Xinjiang.

It has been widely accepted that the well-known historical Silk Road linking Asia, Europe and Africa, had played a key role in material trades, national reconciliation and culture exchanges. There has been growing evidence that some non-formalized long-distance trade and communication connecting the West and East Eurasia could have occurred and were perhaps mediated by nomadic pastoralists well before the historical Silk Road (Proto-Silk Road). The observation of various archaeological cultures belonging to both East and West Eurasia [44,45] and the co-existence of diverse cereals of Near East (e.g. barley and wheat) and East Asia origin (e.g. foxtail and broomcorn millets) in Xinjiang more than 4,000 years before present [46], points out the significant role Xinjiang had played for cultural, cereal and population contacts. This dynamic crossroad could also have provided the possibility for the spread of infectious diseases such as plague, leprosy, anthrax and intestinal parasites along the Proto-Silk Road [22,47–49]. While so far there has not been a study focusing on East Asia and specifically designed to capture ancient *S*. *enterica* genomes, they had been identified from human skeletons in Europe dating since 6,500 BP [6,8]. Furthermore, human genetic studies demonstrated that both East and West Eurasian ancestry was detected in the XBQ individuals [50]. We propose that the emergence of *S*. *enterica* in this region could have been promoted by the frequent contacts between populations from the west and the east along the Proto-Silk Road, which facilitated the spread of this basal lineage that likely led to substantial mortality during Eurasian prehistory. Of course, given the limited ancient data, other possible routes cannot be excluded. It’s necessary to obtain more ancient *Salmonella* DNA to reveal the accurate spreading routes in the future.

Altogether, our study shows that *S*. *enterica* infected human populations in East Asia at least 3,000 years ago, which long before any historical recording of pandemics in China. Our findings contribute to the known genetic diversity of *S*. *enterica* and reveal a previously undetected branch that falls very close to the ancestor of modern Paratyphi C, Typhisuis and Choleraesuis in the Para C lineage. Interpreting our data within the context of phylogenetic position and human population genetic background, we observed epidemic events in Xinjiang, which represents the earliest evidence that the Proto-Silk Road contributed to the spread of infectious diseases, which were likely to explain the substantial mortality in some Eurasian populations during prehistory.

## Materials and methods

### Archaeological context

The XBQ site is located in the Barkol Hakka Autonomous County, Hami District, Xinjiang Province, China (N43°30’ ~ N43°32’, E93°18’ ~ E93°20’, Fig 1). The cemetery was excavated by a joint team consisting of the Hami Bureau of Cultural Heritage, Barkol County’s Cultural Relics Administration and the School of Cultural Heritage conservation of Northwest University in 2008 [23]. More than 200 burials were found in XBQ. In addition to the plant remains such as barley and wheat, some animal skeletons like cattle, sheep, horse and deer were also identified. This indicates that the XBQ population based their subsistence on cereal farming and animal husbandry, with the latter as the main contributor.

### DNA extraction, library construction and shotgun sequencing

Teeth were collected from 14 individuals excavated from XBQ. 50 mg powder per sample was removed using a dental drill following a previous study [18]. Since the preserved pathogen DNA is more likely to reside in the dried blood vessels of the pulp chamber, we only retrieved tooth powder from the dental pulp [51]. All procedures were carried out in the dedicated ancient DNA facilities in Jilin University, China. DNA extraction was performed according to a previously described protocol, with a rotation of 12-16h at 37°C during an initial lysis step [52]. A negative control was included for each step. The extraction resulted in 100 μl of DNA extract per sample. 20 μl were used for library construction as described by Meyer and Kircher [53]. Each sample was indexed with 8bp specific indices and library amplification was performed using Q5 High-Fidelity DNA Polymerase (New England Biolabs). Libraries were cleaned using Agencourt AMPure XP beads (Beckman Coulter) with a ratio ranging from 1:1.5 to 1:1.8 (library volume:bead volume) and were finally eluted in 26 μl sterile water. These libraries were subsequently used for shotgun sequencing. Multiplex shotgun sequencing was carried out using an Illumina HiSeq X10 platform at Novo Inc., Beijing, China in PE 150 mode.

### Screenings methods

We used the MEGAN alignment tool (MALT), a program for the fast alignment and analysis of metagenomic DNA sequencing data [54]. The database, which includes all bacterial genomes in NCBI Refseq (December 2016) was built using the malt-build command. In order to run MALT, 14 samples were first processed with the EAGER pipeline to perform adapter clipping and paired-end read merging [55]. Merged reads were subsequently screened by malt-run. The “minimum percent identity” parameter was set to 90 (-id), the minimum support parameter was set to 1 (-sup), a top percent value of 1 (-top) was set, the maximum number of alignments per query was set to 100 (-mq), BlastN mode and SemiGlobal alignment were applied. The generated rma6 files were visual inspected in MEGAN [56] and were further processed with HOPS [57], an automated java-based pipeline incorporating the metagenomic alignment tool MALT, which focuses on screening rma6 data for the presence of a user-specified list of target species. Specifically, the MALT output (rma6) was analyzed with MALTextract, a newly designed tool within HOPS that allows automatic retrieval of alignment information from rma6 files.

### Probe design, in-solution capture and sequencing

Probes were designed based on publicly available reference sequences including *Salmonella* chromosomes/assemblies and plasmids [7] (S6 Table). Due to the degradation characteristics of ancient DNA, previous studies have shown that a shorter probe length (e.g., 60 bp) works better for ancient DNA, while probes that work efficiently for modern samples can be up to 120 bp in length [58]. In order to enrich *S*. *enterica* genomes in a more cost-efficiency way, we designed the capture probes with a length of 100bp. Through the above design, we produced a total of 92,710 probes with 100 bp tiling. Probes were synthesized by iGeneTech Co. Ltd in China.

The libraries that were identified as positive for *S*. *enterica* were then enriched using an in-solution DNA capture with the probes described above. Before hybridization, the DNA library was amplified used KAPAHiFi HotStart enzyme to reach a concentration of 500ng/μl. RNA probes and adapter blockers were used following the kit manufacturer instructions. The hybridization condition was set to 62° for 48 hours in order to make a full hybridization between the DNA library and the probes. The Dynabeads Myone streptavidin T1 magnetic beads were used for capture because of its strong non-covalent interaction with streptavidin. Lastly, the captured libraries were re-amplified with IS5 and IS6 for 14 cycles. Subsequently, the libraries were purified by AMpure XP beads, quantified by Bioanalyzer 2100 and sequenced on a HiSeq X10 platform.

In order to avoid the incorporation of incorrect bases due to ancient DNA modifications, the positive samples were pre-treated with USER enzyme (New England Biolabs), which contains Uracil DNA Glycosylase (UDG) and endonuclease VIII (endoVIII) [59]. UDG treatment removes uracil residues, which are most common at the 5- and 3-prime end of ancient DNA molecules.

### Read processing, mapping and SNP calling

All samples were processed with the EAGER pipeline [55]. Sequencing quality for each sample was evaluated with FastQC, and adaptors clipped using the AdapterRemoval module in EAGER and reads shorter than 30 bp were discarded [60]. The preserved reads were then mapped with BWA v0.7.17 [61] to Paratyphi C RKS4594 (NC_012125.1) as a reference genome. Non-UDG libraries (BWA parameters: -l 16; -n 0.01; -q 37) were mapped with looser parameters, while UDG treated libraries were mapped with stringent parameters (BWA parameters: -l 32; -n 0.1; -q 37). The duplicates were removed by the DeDup tool implemented in EAGER. The ancient DNA damage patterns were characterized using mapDamage [28].

To increase the depth of data, the raw fastq file including the UDG and Non-UDG data were merged using the zcat command and then processed with the EAGER pipeline using stringent alignment. In order to minimize the bias due to ancient DNA deamination, the final bam was trimmed according to the frequencies of C to T or G to A at both 3’ and 5’ ends to a degree that the damages at the end of the trimmed reads were identical to the baseline. The trimmed reads were performed SNP calling using the UnifiedGenotyper of the Genome Analysis Toolkit (GATK) with high-quality base score (Q ≥30), using the “EMIT_ALL_SITES” option, which generates a call for each genomic site [62].

### SNP evaluation and phylogenetic analyses

In order to infer the phylogenetic placement of the ancient genomes, we compiled a dataset including 475 modern *S*. *enterica* genomes, the 4 previous published ancient *S*. *enterica* genomes [6–8] as well as 6 genomes from this study. All modern genomes were fragmented 100bp with 1bp step size, then aligned to the Paratyphi C genome using BWA with strict parameters (bwa aln -l 32, -n 0.1, -q 37). SNP calling was carried out with the Genome Analysis Toolkit (GATK) UnifiedGenotyper [62] using a genotype quality score of ≥30 for the ancient genomes and the modern genomes, and using the ‘EMIT_ALL_SITES’ option, providing a call for all variant or non-variant bases in the vcf file output [62]. MultiVCFanalyzer [63] was used for collating homozygous SNPs (Minimal genotyping quality set as 30, 90% of reads covering a position must be in agreement) called at a minimum of 3X coverage against the *S*. Paratyphi C RKS4594 reference [63]. SNP alignments were used for phylogenetic analysis. We evaluated SNP calls by using the SNPEvaluation tool [29]. SNPs were called true positive when meeting the following criteria within a 50-bp window: (A) The reads were mapped using stringent parameters (bwa aln -l 32,–n 0.1 and -q 37), (B) no “heterozygous” positions, and (C) no non-covered positions. All false-positive SNPs were discarded for the phylogenetic tree construction.

A maximum likelihood tree was generated by IQ-Tree 1.6.12 [30], which was run using ModelFinder with the option–m MFP. A total number of 478 models were tested, we identified GTR+F+ASC+R3 as the best substitution model. 1,000 fast bootstrap replicates were performed to assess statistical support at each node. The Arizonae serovar was set as outgroup. The result showed that our ancient genomes cluster within the Para C lineage. In order to further verify the phylogenetic tree of our ancient genomes, we constructed another maximum parsimony tree with 500 bootstraps using 219 modern Para C genomes and ancient genomes by the MEGA-proto and executed using MEGA-CC [64].

Given that conventional phylogenetic analysis on low coverage data could produce biased results, we also adopted an additional approach for phylogenetic placement (MGplacer) [31], whereby low coverage genomes were placed on a fixed Maximum Likelihood tree. 219 modern Para C group genomes were used to construct a maximum-likelihood tree, and MGplacer2.py was used to determine the branch location of our ancient genomes. Then our low coverage data were plotted on the fixed tree. The generated tree was visualized using FigTree v1.4.3 [65] (http://tree.bio.ed.ac.uk/software/figtree/) and Evolview [66] (https://evolgenius.info/evolview-v2).

### Estimation of divergence times

The SNP alignment was used for molecular dating with BEAST v1.10.1 [67]. Before this, we investigated if the data shows a temporal signal using TempEst v1.5.1 [34]. Ancient genomes XBQM20 and XBQM90 were selected for the dating analysis as they showed sufficient genomic coverage. Modern Para C genomes, and the ancient genomes Tepos14, Tepos35 and ragnaU were included. A root to tip regression of genetic distances and sampling times showed a sufficient temporal signal and permitted us to proceed with the molecular dating analysis. For tip dating, all modern genomes were set to an age of 0, ancient genomes were set according to their C14 dating (2-sigma range). We used a relaxed lognormal molecular clock, with the GTR+G model of nucleotide substitution, with a discrete gamma distribution and six rate categories to account for rate heterogeneity across sites applied with the Bayesian skyline coalescent model. The generated XML file was run by BEAST. For our dataset, 100 million MCMC steps were computed sampling every 5,000 steps. The output log file was analyzed with Tracer with a 10 percent burn-in. A maximum clade credibility tree with a 10% burn-in was produced using the TreeAnnotator [67]. The tree was visualized with FigTree v1.4.3 [65].

### Presence/absence analysis of *S*. *enterica* virulence factors (SPI, virulence plasmid and phages)

We collected annotations for SPI-1 through SPI-16 as well as for the virulence plasmid and phages from the PAIDB database and previous studies [6, 68–74]. The plasmid annotation files were downloaded from NCBI. Each SPI as well as plasmid was used as a reference for mapping all reads from ancient and modern genomes part of the Para C lineage with BWA mem [75]. Alignments were filtered using Picard Tools (CleanSam, MarkDuplicates) [75]. Read depth for each gene was summarized using bedtools (bedtools coverage -a file.bed -b bamfile > depth.txt) [76]. We considered only genes that had at least 95% of its sequence covered by the capture probes. Heatmaps were plotted using the ggplot2 package in R [77].

### Pseudogene analysis

Pseudogenes are coding sequences (CDS) that are putatively inactivated by mutations including nonsense substitutions, frameshifts, or truncation by deletion or rearrangement [78]. Here, we infer the frequency of pseudogenes per genome similar to Key et al. [8]. Briefly, we use a pangenome of *S*. *enterica* based on all CDS from 537 representative genomes, providing 21,065 genes after filtering and cleaned for paralogs. We included only genes 100% covered by the probe set used for capture, leading to 8,726 genes used for analysis. All genomes from the Para C Lineage were included together with additional *S*. *enterica* genomes with known host specificity. We inferred pseudogenes using GenCons [79] to build a consensus sequence using the following two criteria: we reported every position with allele and total coverage of at least 1X and an alternative allele frequency of at least 66% (thus SNPs are only called with at least two alternative alleles in agreement). We infer frequency of pseudogenes per genome using all genes that have an allele called in at least 90% of positions.

## Supporting information

S1 Fig HOPS screening results for *S*. *enterica* positive samples. The edit distance distribution represented the number of nucleotide positions in a mapped DNA sequence that differ from the reference that it aligns to, eg. if there is one mismatch in the alignment the edit distance is 1. The image on the left: Edit distance distribution for all reads assigned to *S*. *enterica*. The image on the right: Edit distance distribution for assigned reads that show a damage signal.
(TIF)

S2 Fig HOPS output of DNA damage plot for assigned *S*. *enterica* reads. C-to-T and G-to-A substitutions from the 5’ end and the 3’ end were presented in our 6 positive samples, which is typical for ancient DNA.
(TIF)

S3 Fig Mismatch distribution along positions at the 5’- and 3’- end of mapped sequencing reads. C to T changes indicated in red and G to A changes in blue, all other substitutions in grey.
(TIF)

S4 Fig Maximum Likelihood tree of modern Salmonella Para C lineage and XBQ strains. 219 modern Para C group genomes were constructed a maximum-likelihood tree, XBQ data were mapped to the tree with MGplacer. As a result, XBQ strains were placed between the node Br_219 and Br_220, which were basal to the position of the Paratyphi C, Typhisuis and Choleraesuis.
(TIF)

S5 Fig The read depth at nucleotide position of SPI-7 in XBQM20. (TIF)

S6 Fig Root-to-tip regression analysis. Plots of the root-to-tip genetic distance against sampling time were shown. All modern genomes were set to an age of 0, ancient genomes were set according to their C14 dating. Sampling dates were given as years before the present. The dataset yielded R^2^ of 0.2368, which confirms the existence of temporal signal.
(TIF)

S7 Fig Maximum Clade Credibility tree. The MCC tree was produced using TreeAnnotator of BEAST v1.10.1. The tree was visualized in FigTree v1.4.3 (http://tree.bio.ed.ac.uk/software/figtree/). It is presented in a temporal scale between 25,000 and 0 yBP, and the main internal node dates of the Para C lineage are indicated on each corresponding node.
(TIF)

S8 Fig Proportion of shared pseudogenes between strains across the Para C lineage and Birkenhead. Proportion of pseudogene-sharing (0–100%) between strains shown in tones of red. Strains are grouped by serovar.
(TIF)

S1 Table *Salmonella enterica* capture efficiency (capture data have been merged UDG data and Non-UDG data). (XLSX)

S2 Table Bayesian posterior estimates of the time to the most recent common ancestor and substitution rates for the sub-lineage of Para C lineage. (XLSX)

S3 Table Pseudogenes identified in XBQM20S and XBQM90S. Pseudogenes shared between both ancient genomes are marked in yellow.
(XLSX)

S4 Table Information about variant positions detected in one or both of the ancient genomes (XBQM20 and XBQM90). (XLSX)

S5 Table Information about variant positions detected in one or both of the ancient genomes. (XLSX)

S6 Table Accession number information about our probes designed based on publicly available reference sequences including Salmonella chromosomes/assemblies and plasmids. (XLSX)
