# Supplementary figures and images for: A 3,000-year-old, basal S. enterica lineage from Bronze Age Xinjiang suggests spread along the Proto-Silk Road
Source: PLoS Pathog. 2021 Sep 21;17(9):e1009886. doi: 10.1371/journal.ppat.1009886 (PMC8486138; doi:10.1371/journal.ppat.1009886)

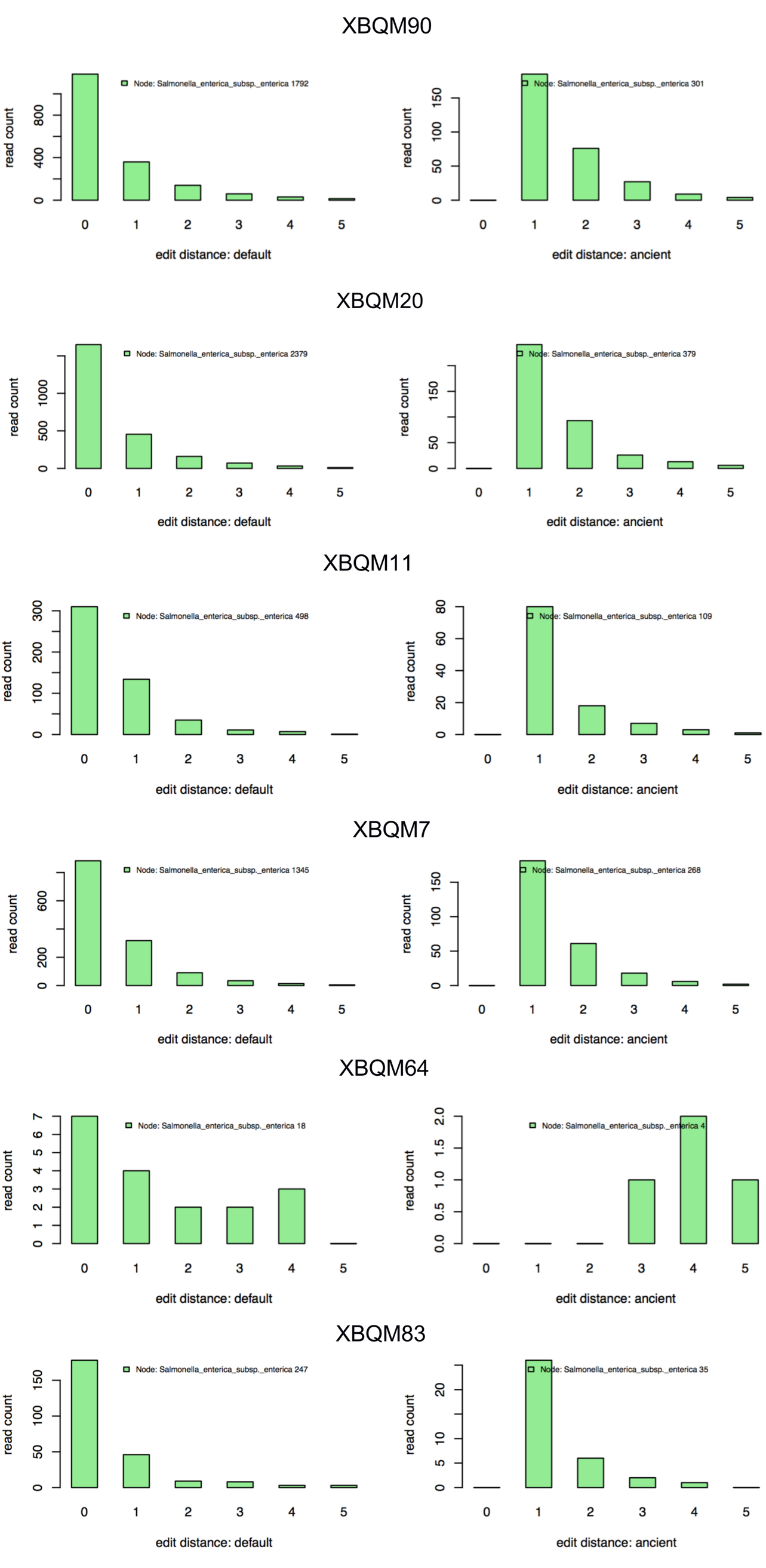

Supplement: S1 Fig — The edit distance distribution represented the number of nucleotide positions in a mapped DNA sequence that differ from the reference that it aligns to, eg. if there is one mismatch in the alignment the edit distance is 1. The image on the left: Edit distance distribution for all reads assigned to S. enterica. The image on the right: Edit distance distribution for assigned reads that show a damage signal. (TIF) [file ppat.1009886.s001.tif]

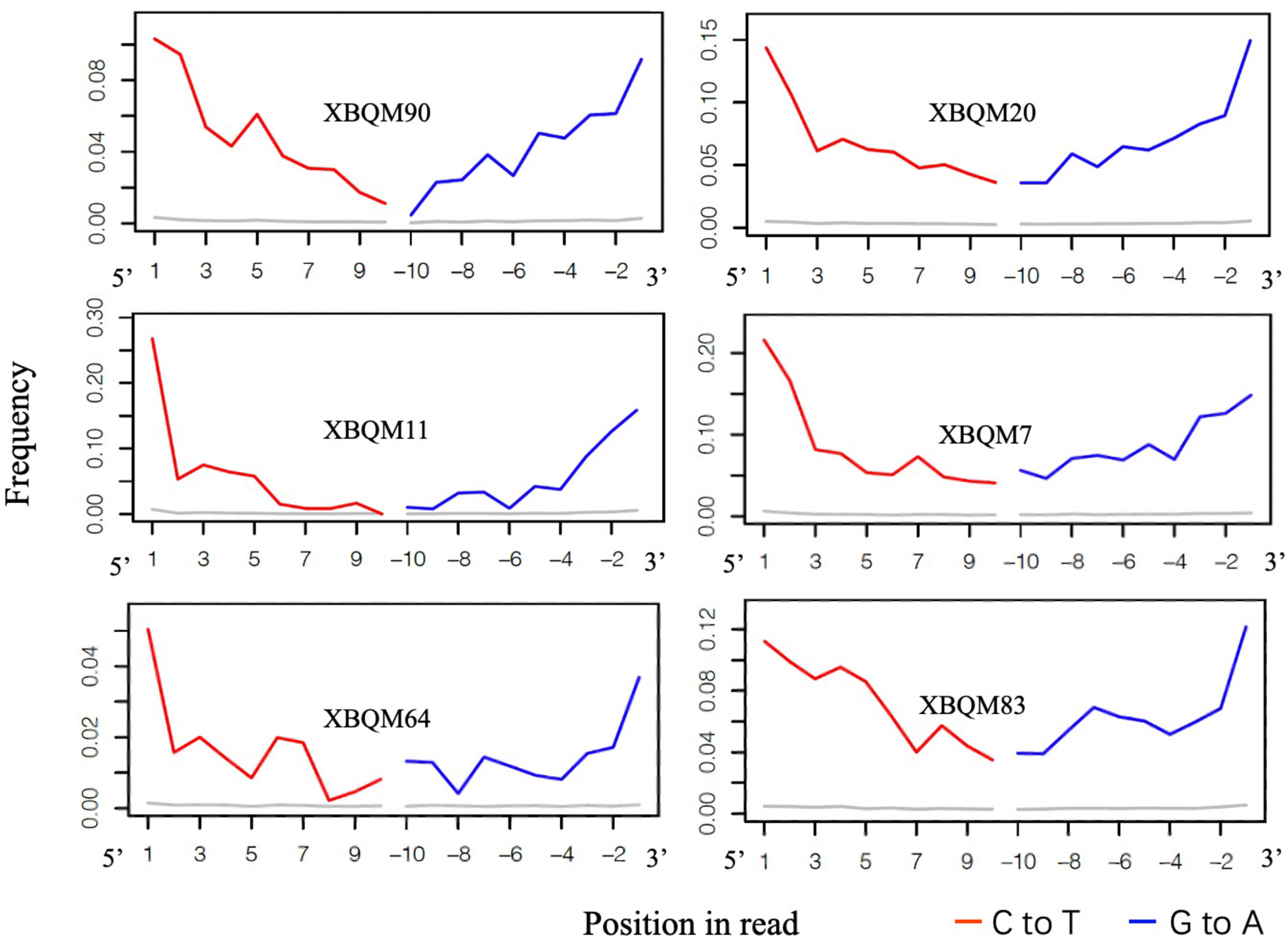

Supplement: S2 Fig — C-to-T and G-to-A substitutions from the 5’ end and the 3’ end were presented in our 6 positive samples, which is typical for ancient DNA. (TIF) [file ppat.1009886.s002.tif]

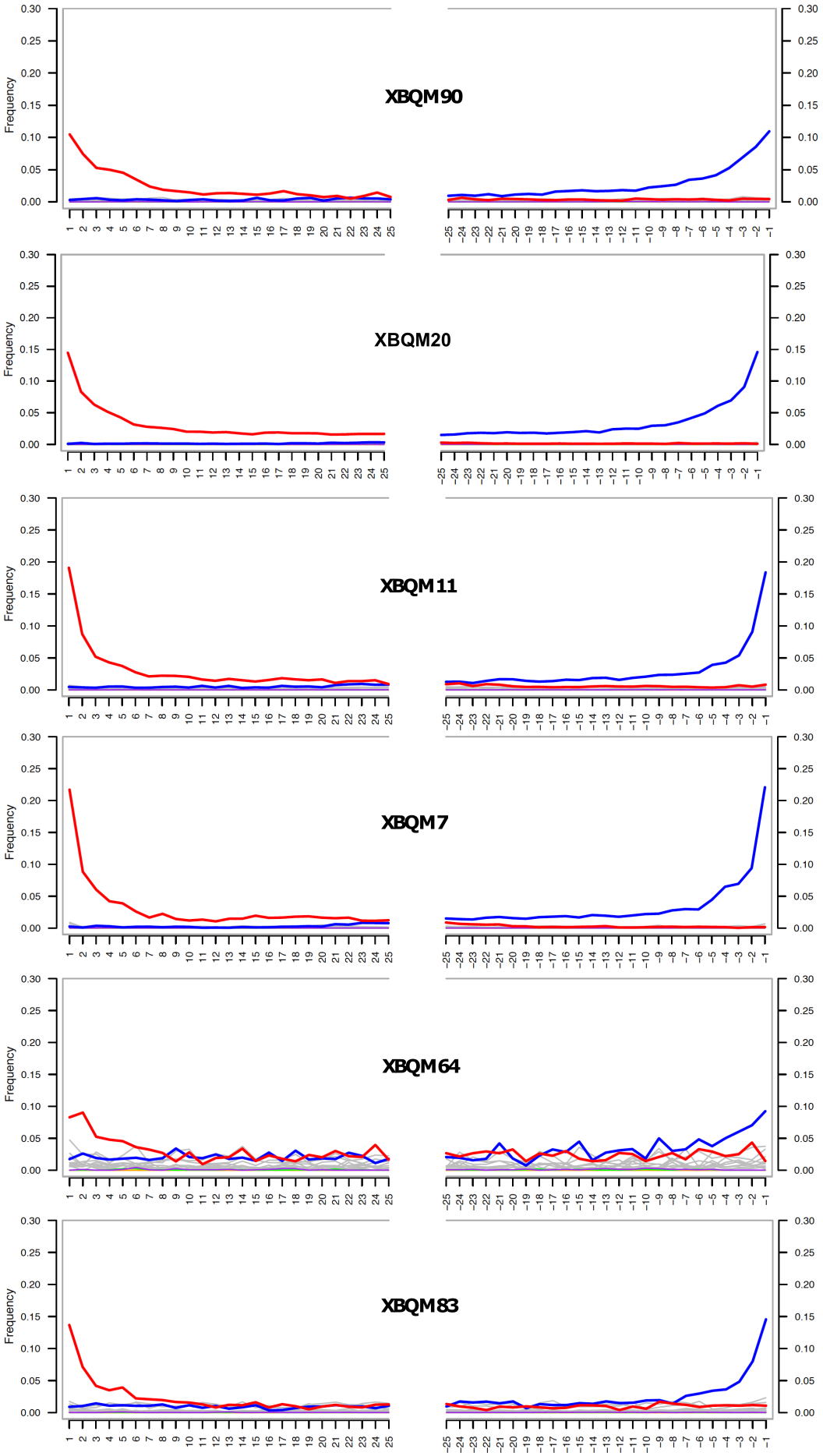

Supplement: S3 Fig — C to T changes indicated in red and G to A changes in blue, all other substitutions in grey. (TIF) [file ppat.1009886.s003.tif]

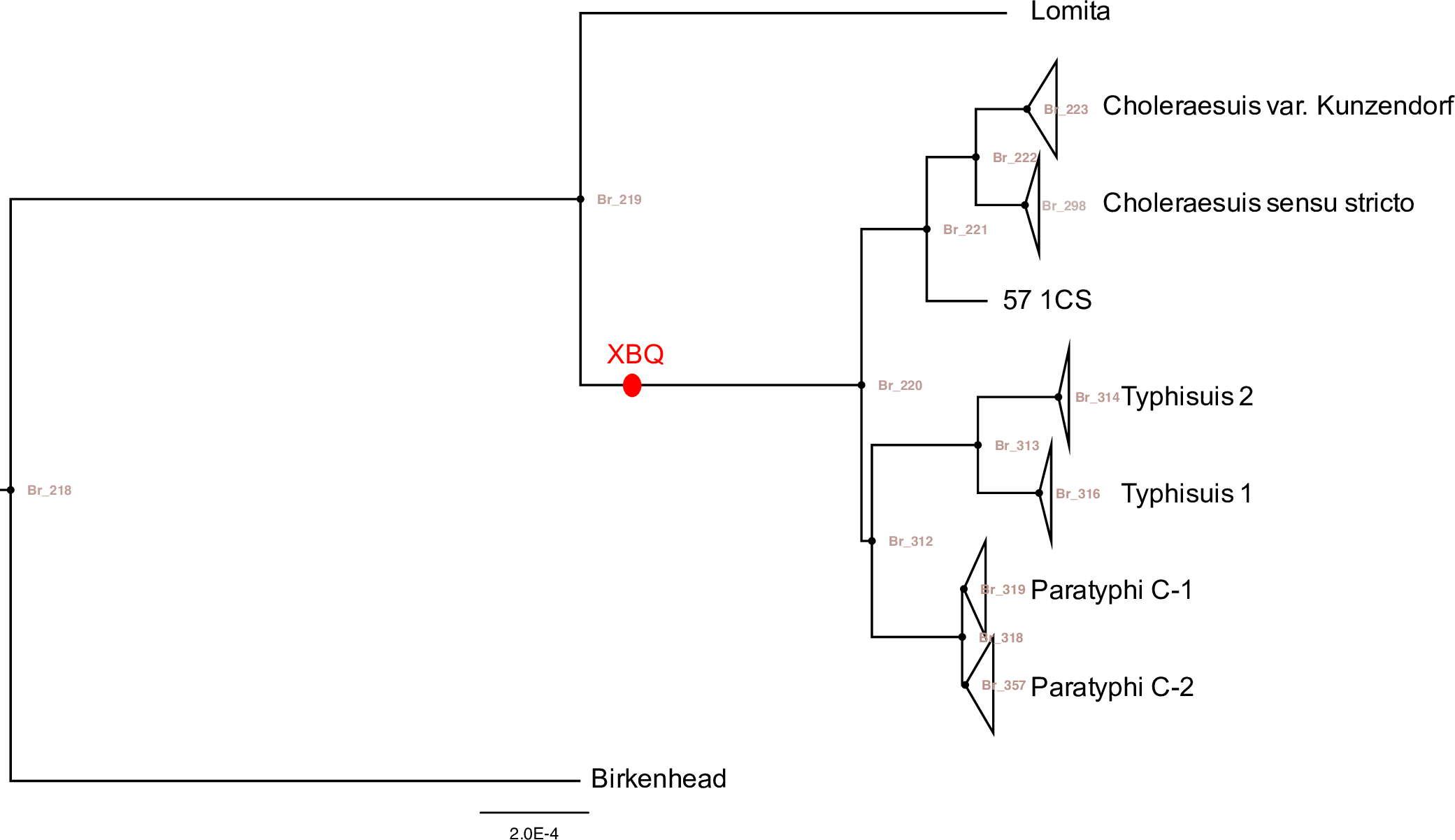

Supplement: S4 Fig — 219 modern Para C group genomes were constructed a maximum-likelihood tree, XBQ data were mapped to the tree with MGplacer. As a result, XBQ strains were placed between the node Br_219 and Br_220, which were basal to the position of the Paratyphi C, Typhisuis and Choleraesuis. (TIF) [file ppat.1009886.s004.tif]

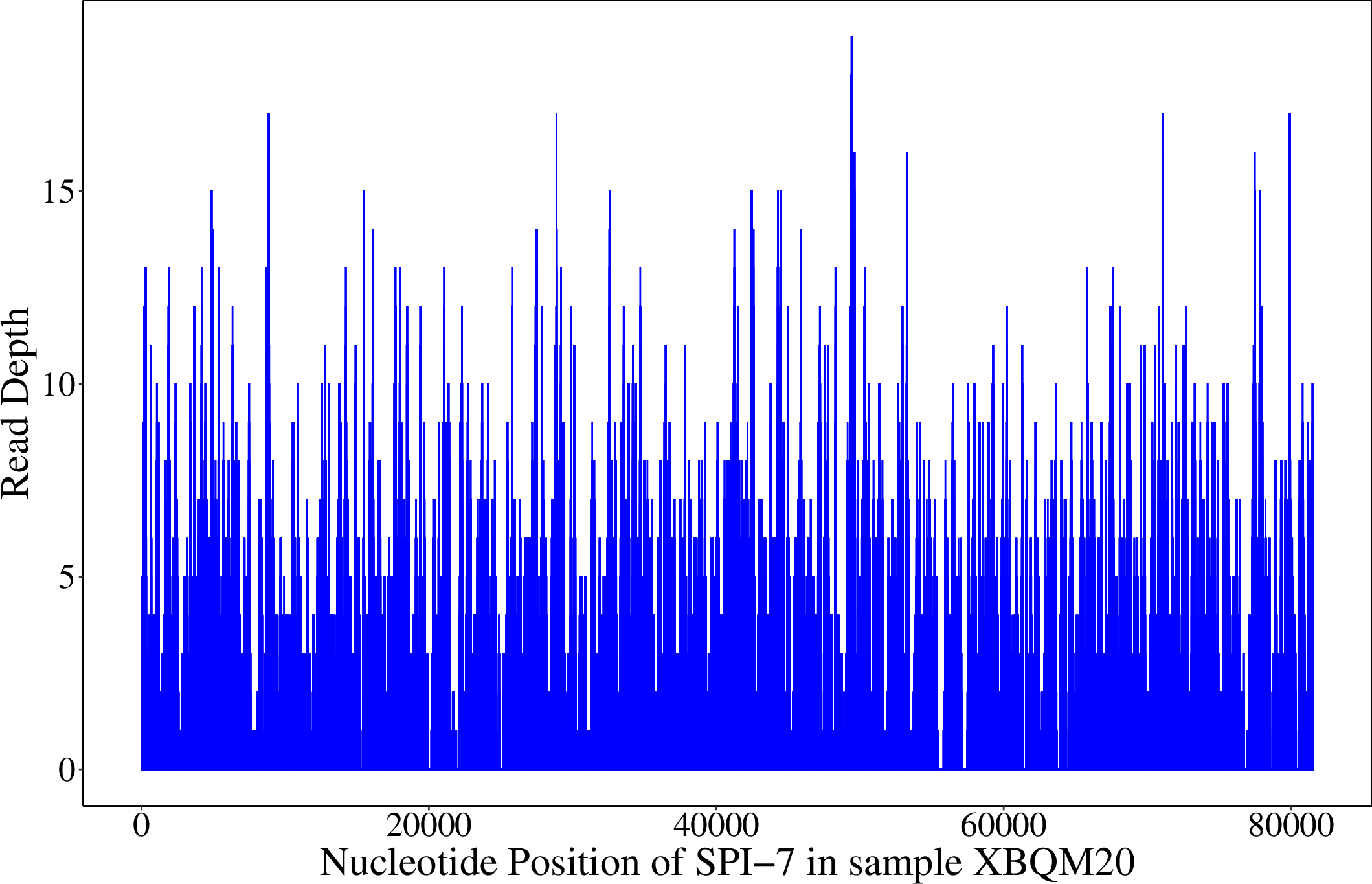

Supplement: S5 Fig — (TIF) [file ppat.1009886.s005.tif]

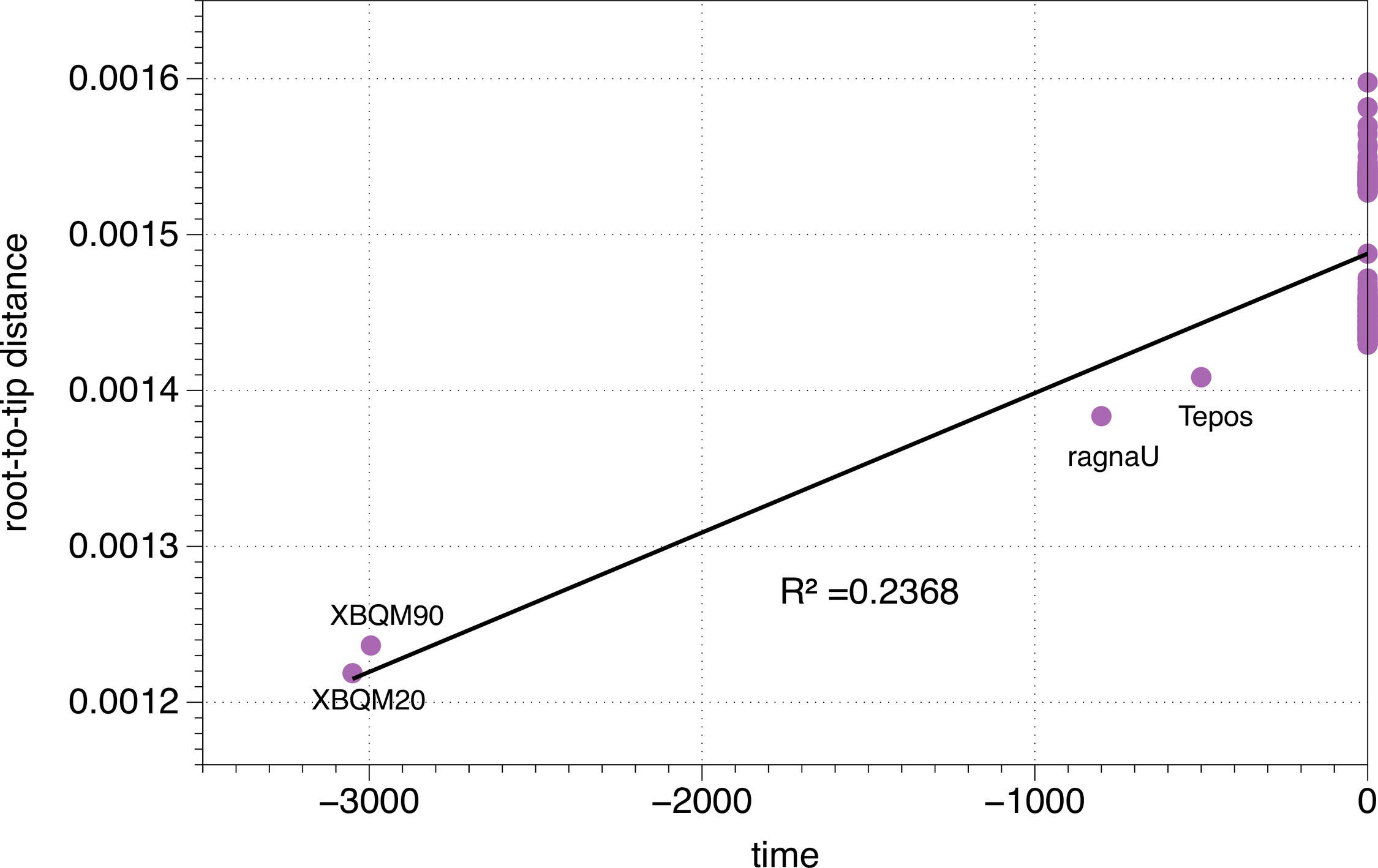

Supplement: S6 Fig — Plots of the root-to-tip genetic distance against sampling time were shown. All modern genomes were set to an age of 0, ancient genomes were set according to their C14 dating. Sampling dates were given as years before the present. The dataset yielded R2 of 0.2368, which confirms the existence of temporal signal. (TIF) [file ppat.1009886.s006.tif]

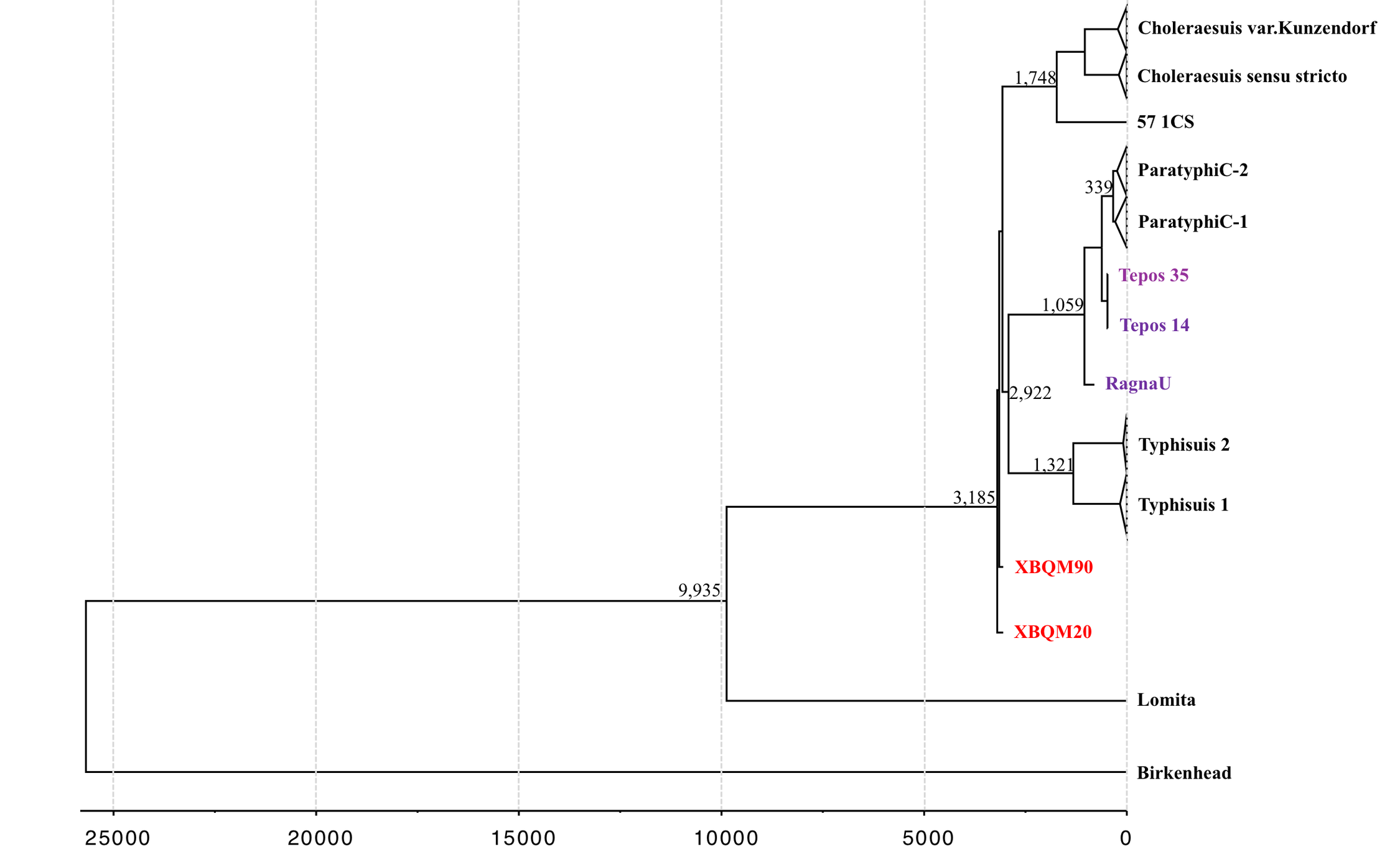

Supplement: S7 Fig — The MCC tree was produced using TreeAnnotator of BEAST v1.10.1. The tree was visualized in FigTree v1.4.3 (http://tree.bio.ed.ac.uk/software/figtree/). It is presented in a temporal scale between 25,000 and 0 yBP, and the main internal node dates of the Para C lineage are indicated on each corresponding node. (TIF) [file ppat.1009886.s007.tif]

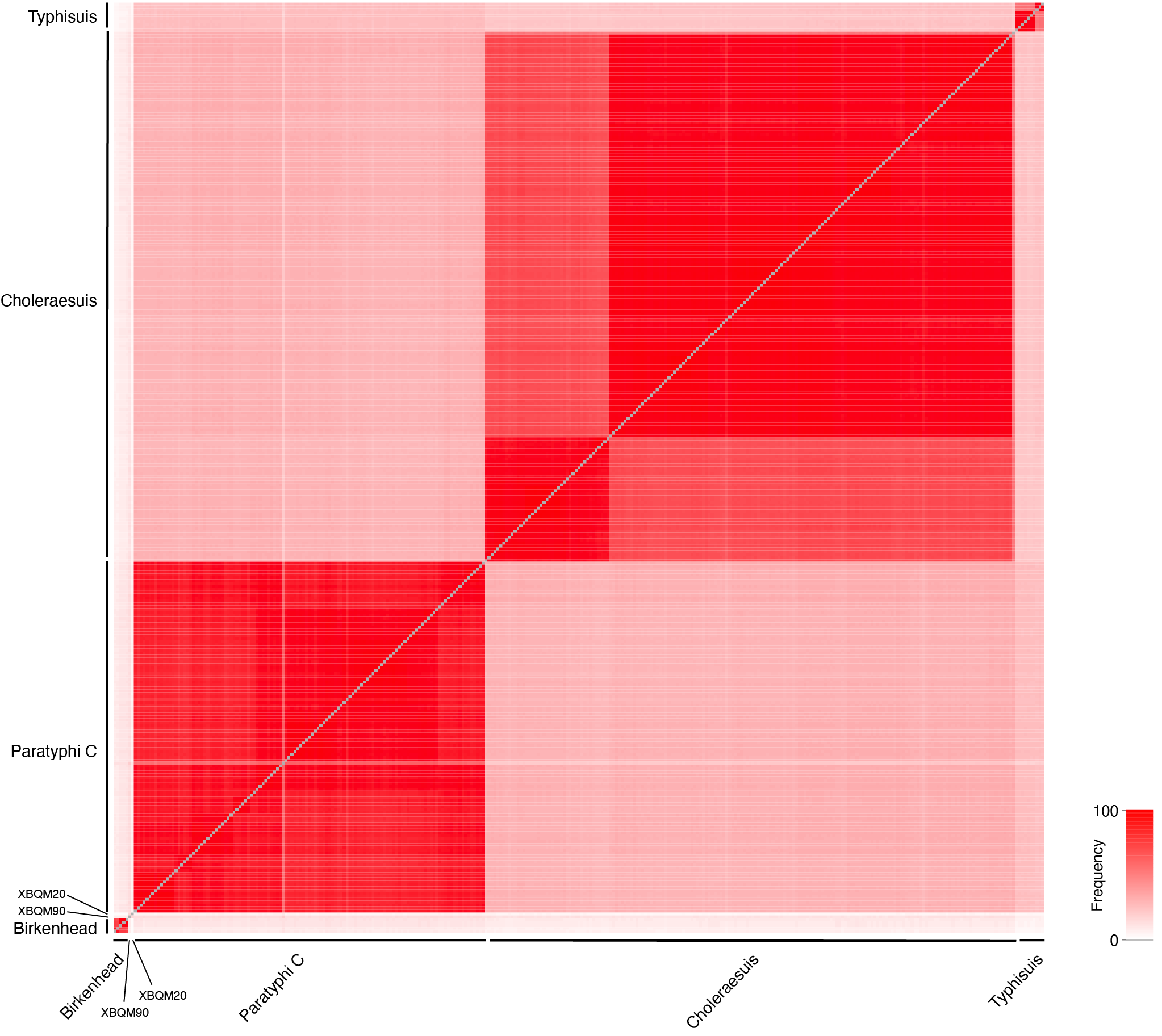

Supplement: S8 Fig — Proportion of pseudogene-sharing (0–100%) between strains shown in tones of red. Strains are grouped by serovar. (TIF) [file ppat.1009886.s008.tif]
